# Immunomodulatory and anti-inflammatory effects of agave fructans in atopic dermatitis: gut microbiota and short-chain fatty acid implication

**DOI:** 10.3389/fimmu.2025.1700023

**Published:** 2025-12-02

**Authors:** Marcela Rios-Carlos, Mariela Jiménez, Daniel Cervantes-García, Laura Elena Córdova-Dávalos, Lidia E. Verduzco, Francisco Javier Enríquez-Medrano, Omar Fabela-Sánchez, Luis G. Bermúdez-Humarán, Eva Salinas

**Affiliations:** 1 Laboratory of Immunology, Department of Microbiology, Autonomous University of Aguascalientes, Aguascalientes, Mexico; 2 Secretariat of Science, Humanities, Technologies and Innovation (SECIHTI), Mexico City, Mexico; 3 Department of Sustainable and Protected Agriculture, Technological University of Northern Aguascalientes, La Estación Rincón, Rincón de Romos, Aguascalientes, Mexico; 4 Department of Macromolecular Chemistry and Nanomaterials, Center for Research in Applied Chemistry (CIQA), Saltillo, Mexico; 5 Micalis Institute, Université Paris-Saclay, INRAE, AgroParisTech, Jouy-en-Josas, France

**Keywords:** atopic dermatitis, agave fructans, prebiotic, short-chain fatty acids, microbiota

## Abstract

**Introduction:**

Atopic dermatitis (AD) is a chronic inflammatory skin disorder resulting from the interplay of genetic and environmental factors, with a dysregulated type-2 immune response. The association between AD onset and intestinal dysbiosis supports research into nutritional interventions such as fermentable fibers intake. Agave-derived fructans (AFs) display prebiotic activity, modulating gut microbial communities that may positively influence immune functions. In this study, we evaluated the anti-inflammatory and immunomodulatory effects of oral AFs in a rat AD model.

**Methods:**

AD-like lesions were induced in the ear of Wistar rats by frequent application of 2,4-dinitrochlorobenzene (DNCB). AFs (0.1, 1, 5 g/kg) from *Agave tequilana* Weber var. azul were orally administered for 13 days. Inflammation, pruritus, gene expression of transcriptional factors of immune response, and staphylococcal colonization were evaluated in lesional skin. Cytokine expression, relative abundance of the main bacterial phyla and genera, and levels of short-chain fatty acids were analyzed in the intestinal milieu.

**Results:**

Treatment with AFs at 0.1 g/kg significantly reduced ear thickness at 1- and 6-hours post-DNCB application. Similarly, ear edema at 1 hour was attenuated, and inhibition of the NF-κB inflammatory pathway was detected. After AFs treatment at 0.1 g/kg, serum IgE levels were normalized to those of control animals. All AFs significantly decreased dermal mast cell and eosinophil counts, as well as epidermal thickening, with greater efficacy observed at lower doses. The scratching behavior remained unchanged across groups. AFs reduced *Staphylococcus aureus* abundance in lesional skin and restored *Staphylococcus epidermidis* levels to baseline. In lesional tissue, AFs downregulated *Gata3*, *Rorc*, *Il4*, and *Il17a* mRNA expression, while promoting a regulatory immune profile in mesenteric lymph nodes, characterized by increased *Foxp3*, *Il10*, and *Tgfb1* expression. Administration of AFs at 0.1 and 1 g/kg promoted fecal abundance of *Bifidobacterium* and cecal acetic acid concentrations, whereas doses of 1 and 5 g/kg upregulated Firmicutes, *Lactobacillus*, and propionic acid levels. All doses reduced Proteobacteria abundance.

**Conclusion:**

AFs exhibit anti-inflammatory, immunoregulatory, and microbiota-modulatory properties in both the gut and skin compartments, in a non-linear dose-response manner. These findings suggest that the intake of AFs may contribute to the therapeutic management of AD.

## 1 Introduction

Atopic dermatitis (AD) is the most prevalent chronic inflammatory skin condition (1). It affects approximately 2.2% of the global population (2), with a prevalence of 13% in children and 5% in adults (3). AD is characterized by eczematous skin lesions, intense pruritus, and epidermal barrier dysfunction, including microbial dysbiosis (4). This disease has a multifactorial etiology involving genetic, immunological, and environmental factors that compromise skin barrier integrity and facilitate allergen penetration (5). In response to epithelial damage, keratinocytes release alarmins that initiate a type-2 immune response, characterized by the activation of innate lymphoid cells (ILC)2, IgE overproduction by plasma cells, recruitment of eosinophils and mast cells, and differentiation of T cells toward a TH2 profile. A type-2 inflammatory cascade dominates the early phase of the disease, with acute lesions extensively infiltrated by cells expressing IL-4 and IL-13 cytokines (6, 7). As the disease progresses, type-1 immune cells and cytokines are involved, contributing to the chronicity of the disease (8). T-cells producing IL-17 and IL-22 have also been implicated in the initiation and maintenance of AD (8, 9). Recent evidence shows that type-1, -2, -17 and -22 responses participate in both acute and chronic inflammation in AD, with the transition between disease stages being quantitative rather than qualitative in terms of cytokine network dynamics (10). In addition, the alterations in the skin microbiota, such as increased staphylococcal abundance and exacerbated colonization by *Staphylococcus aureus*, contribute to lesion severity and modulate the expansion of specific TH cell profiles (11).

The gut microbiota is another key element in the regulation of the immune response in AD (12). Indeed, commensal bacteria residing in the distal part of the mammalian intestine contribute to host health by facilitating digestion, synthesizing vitamins, promoting the development of gut-associated lymphoid tissues, modulating gut-specific immune responses, and preventing pathogen colonization (13). In addition, the beneficial effects of intestinal eubiosis extend to distal organs, such as the lungs and skin. Thus, multiple cohort studies suggest that gut microbiota dysbiosis precedes the onset of atopic diseases (14, 15). One of the main mechanisms linking gut microbiota to skin homeostasis involves short-chain fatty acids (SCFAs), which are major immunomodulatory metabolites generated by microbial fermentation of dietary fiber and carbohydrates in the colon (16, 17). Nevertheless, not all fermentable fibers have the same capacity to stimulate SCFA production or induce shifts in the colonic microbial community (18).

Agave fructans (AFs), also known as agavins, are potential carbohydrate substrates for intestinal microbiota. They are polysaccharides consisting predominantly of repeating fructose units linked by β(2→1) and β(2→6) bonds, with an internal glucose molecule and a branched structure (19, 20). Fructans act as reserve carbohydrates in plants of the *Agave* genus, enabling them to withstand adverse environmental conditions such as drought and extreme temperatures. Among these, *Agave tequilana* Weber var. azul is the most recognized and economically significant species in Mexico, as it is the sole taxon authorized for tequila production under the designation of origin (21). AFs are soluble fibers with prebiotic properties. Upon ingestion, they remain undigested in the upper gastrointestinal tract and reach the colon intact, where they promote the growth of beneficial bacteria, such as *Bifidobacterium* and *Lactobacillus* (22). This prebiotic effect contributes to intestinal health and can positively modulate the immune system (23, 24). However, the impact of fructans on the microbiota is not uniform, studies indicate that their prebiotic efficacy depends on the degree of polymerization (DP) (25–27), which, in the case of AFs varies according to species, geographic origin, and plant age (19).

The effects of fructan-type fibers on experimental allergies have been studied mainly using chicory inulin (CI). Inulin intake by pregnant rats modulates the intestinal microbiome composition in both mothers and offspring, contributing to attenuated asthmatic inflammation in offspring (28). In addition, oral administration of inulin to mice with AD reduces lesion severity and the skin inflammatory response (29, 30). However, there are conflicting findings indicating that dietary inulin may exacerbate type-2 inflammation in mice (31). Notably, inulin fructans consist of linear fructose chains linked by β (2→1) glycosidic bonds and exhibit a DP that is distinct from that of AFs (32). Consequently, their fermentation by the gut microbiota is different, and their biological effects are not generally comparable to other fructans. Thus, the present study aimed to evaluate whether the oral consumption of AFs from *A. tequilana* Weber var. azul exerts anti-inflammatory and immunomodulatory effects in an AD-model in rats, as well as to decipher the underlying mechanisms.

## 2 Materials and methods

### 2.1 Animals

Wistar rats (male, 110–130 g of body weight) were acquired from the Laboratory Animal Service of the Autonomous University of Aguascalientes and housed under conventional standardized conditions, with temperature (22–24 °C) and light (12-hour light–dark cycle) controlled. Male rats were selected to establish the AD model, since males have been widely employed in previous AD studies (33–35). The rats had free access to food (rodent chow diet, Nutricubo, Purina, USA) and water. All animal experiments were approved by the Institutional Ethical Committee for the Use of Animals in Research (Approval code: INV 012/2023) and complied with the institutional guidelines for experimental animal care and the national regulatory norm (NOM-062-ZOO-1999).

### 2.2 Induction of experimental atopic dermatitis

**Figure 1:**
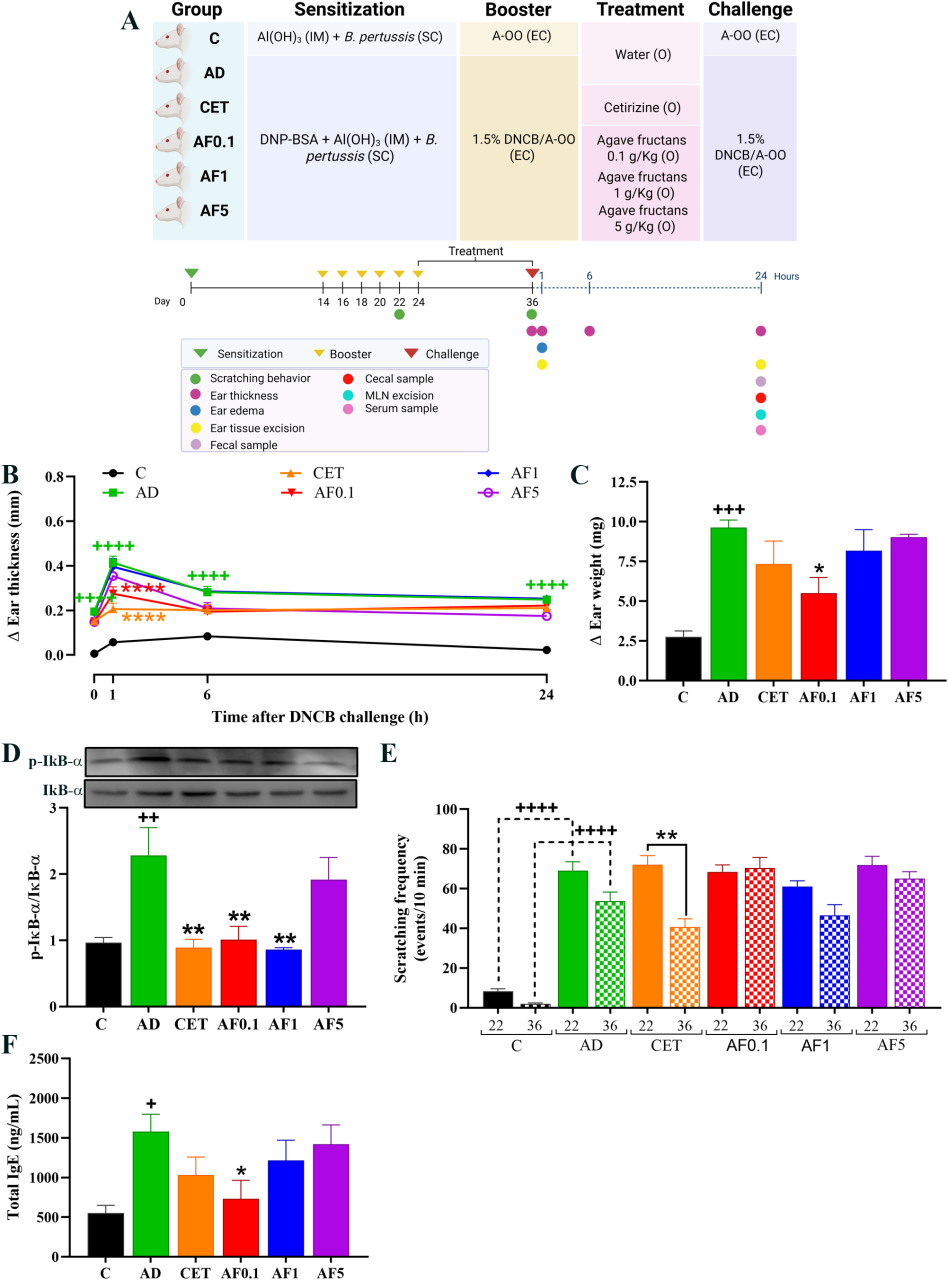
Experimental model and agave fructans effect on clinical signs of atopic dermatitis in rats. **(A)** Schematic representation of experimental atopic dermatitis induction and experimental design. The first sensitization of the animals was on day 0 with intramuscular (IM) injection of DNP-BSA mixed with Al(OH)_3_ gel and simultaneously with *B. pertussis* vaccine subcutaneous (SC). Animals were re-sensitized (boosted) with epicutaneous (EC) application of DNCB in acetone-oil olive (A-OO) on days 14, 16, 18, 20 and 22. Treatments were administered orally (O) after atopic dermatitis induction, daily, from day 24 to day 36. Clinical signs were analyzed, or samples were collected on day 36 after the last DNCB challenge. **(B)** Skin inflammation in lesional tissue before (0h), one, six and 24 hours after last DNCB challenge; n=8 rats. **(C)** Ear edema and **(D)** and NF-kB activation 1 hour after DNCB application on day 36; n=4 rats. **(E)** Scratching events induced by DNCB in animals on days 22 and 36; n=8 rats. **(F)** Serum total IgE levels 24 hours after DNCB challenge; n=8 rats. +P < 0.05; ++P < 0.01; +++P < 0.001; ++++ P < 0.0001 vs. C; *P < 0.05; **P < 0.01; ****P < 0.0001 vs. AD.

After one week of acclimatization, AD was induced in the animals by frequent applications of 2,4-dinitrochlorobenzene (DNCB; Sigma, St. Louis, MO, USA) on the skin of the ear after systemic sensitization, as previously described (33, 36). Animal sensitization (day 0) was carried out by intramuscular injection of 10 µg of dinitrophenyl-bovine serum albumin precipitated in 7.8 mg of aluminum hydroxide gel (Thermo Scientific, Waltham, MA, USA) dissolved in 1 mL of saline solution; at the same time, as an adjuvant, 0.5 mL of DPT vaccine (inactivated *Bordetella pertussis* 4 IU, Diphtheria toxoid 30 IU, Tetanus toxoid 60 IU, DIPERTIX; Biofarma, Bandung, Indonesia) was injected subcutaneously. On days 14, 16, 18, 20 and 22, the animals were re-sensitized with a topical application of 60 μL of 1.5% w/v DNCB prepared in an acetone-olive oil (A-OO) vehicle (4:1) to both sides of the right ear lobe of rats. Repeated DNCB application generated an inflammatory process in the right ear of the animals (**Supplementary Figure S1A**). The control group was injected with adjuvants only, and the A-OO solution was applied topically. On day 36, all animals received a final challenge with DNCB on the right ear (**Figure 1A**). Animals were weighed on days 0, 7, 14, 22, 28 and 36 of the protocol.

### 2.3 Experimental design and sample collection

Animals were randomly assigned to six groups of 12 rats each: C, not sensitized; AD, sensitized with DNCB to induce AD-like lesions; CET, sensitized with DNCB and treated with 0.2 mg/kg cetirizine (anti-H1 histamine receptors; Zyrtec, Amstrong Laboratories, CDMX, México); AF0.1, sensitized with DNCB and treated with AFs at 0.1 g/kg; AF1, sensitized with DNCB and treated with AFs at a dose of 1 g/kg; AF5, sensitized with DNCB and treated with AFs at a dose of 5 g/kg. The treatments were administered orally and daily, on days 24 to 36, and dissolved in purified water using an esophageal catheter (**Figure 1A**). The control and AD groups were administered with purified water. AFs were obtained from Enature (Zapopan, Jalisco). Each 100 g of this product contains 89 g of agavins (soluble fiber), 8 g of sugar, and calcium (11% of RDI), according to the information provided by the supplier.

On day 36 and one hour after DNCB application, four rats from each group were euthanized using overdoses of inhaled Sevoflurane administered via chamber, and once the absence of vital signs was verified in animals, the ears were dissected for edema and NF-κB activation evaluation. Eight rats of each group were used to measure ear thickness and scratching events, and after 24 h of DNCB application blood samples were obtained under deep anesthesia with inhaled Sevoflurane and then euthanized as described above. Four rats were used to obtain fecal samples for DNA extraction and tissue from the right ears for DNA extraction and morphometric analysis. The remaining animals were used for RNA extraction from mesenteric lymph nodes (MLN) and tissue of right ears, and to obtain cecal content for SCFA analysis.

### 2.4 Ear thickness measurement

The thickness of the right and left ears of the rats was measured three times in the middle area of the ear using a digital Vernier caliper (POWERFIX). Thickness was measured before (0 hour) and 1, 6 and 24 hours after DNCB application on day 36. The increase in thickness was calculated using the following formula: right ear thickness – left ear thickness.

### 2.5 Ear edema evaluation

On day 36, 1 hour after DNCB application, both ears were dissected, and identical portions were extracted from the middle area of the ears with a metallic punch and weighed on an analytical balance (Precisa XT220A, Switzerland) to calculate the edema as follows: right ear weight – left ear weight.

### 2.6 Scratching behavior analysis

On days 22 and 36, after DNCB application, the rats were placed in separate methacrylate chambers (14cm x 24cm x 26cm; W x D x H) with a behind-placed mirror to allow an unobstructed view and were video recorded. The first 5 minutes of recording were discarded as the animals were not fully recovered from anesthesia, and the scratching events were counted in the following 10 minutes. The videos were watched by three blinded observers, and the number of scratching events was counted and represented as scratching frequency. One scratching event was considered as the series of one or more scratching movements by the hind paw directed toward the right ear and ended when the animal either licked its hind paw or placed it back on the floor (33, 37).

### 2.7 Quantification of total IgE

Serum samples obtained by venous puncture were stored at -70 °C until used for IgE determination. Total serum IgE levels were quantified using a rat IgE ELISA kit (Abcam, Cambridge, UK) according to the manufacturer’s instructions, and the results of the colorimetric reaction were read at 450 nm in an iMark microplate spectrophotometer (BIO RAD, Hercules, California, USA).

### 2.8 Histological examination

For histological analysis, ears from three rats per group were randomly selected, fixed in 10% neutral formalin for 24 hours and embedded in paraffin. Cross-sections of 5 µm-thick were stained with hematoxylin/eosin to visualize the epidermis, blue toluidine (0.015 M in 0.2 M citric acid) to identify mast cells or Harris hematoxylin/erythrosin B (0.15% in 0.1M glycine, pH 10) to identify eosinophils. For epidermal thickness measurement, six photos per histological section were taken (three on the right side and three on the left side of the ear cartilage). In each photo, three measurements of the thickness of the epidermis were made (upper, middle and lower parts of the photo). For the mast cell count, three photos were taken per section (at the top, middle and bottom of the slice), an area of 40,000 µm^2^ of the dermis was marked out, and the mast cells were counted. Eosinophils were counted in the dermis of each entire histological section. All photographs were taken with A BA310 microscope (Motic, Hong Kong) at 400x with a camera attached to the C-B10 microscope (OPTIKA, Italy) and analyzed using the LITEVIEW program by two evaluators who were unaware of the experimental group to which each sample belonged.

### 2.9 Western blot analysis

A portion of the ear tissue was immediately frozen in liquid nitrogen after dissection. Frozen tissue was manually homogenized with a ceramic pestle, suspended in PBS and centrifuged at 417 xg for 5 minutes at 4 °C. Proteins from the supernatant were extracted using lysis buffer with protease inhibitors, and centrifugation at 16,435 xg for 20 minutes at 4 °C. Supernatant proteins were quantified using the Bradford method, and 50 μg of total proteins were separated by 16% acrylamide gels under denaturing electrophoresis at 90 volts for 2 hours. Then, proteins were transferred to PVDF membranes at 100 mAmp for 2 hours at room temperature (RT). After a blocking step with TRIS buffer saline (TBS: Tris 20 mM, NaCl 137 mM, pH=7.6) with 3% bovine serum albumin (BSA) for one hour at RT, membranes were incubated with p-IkB-α at 1:1000 (Sc8404, Santa Cruz) prepared in TBS-BSA, at 4 °C, overnight. They were washed twice for 5 minutes with TBS-0.5% Tween (TBST) and incubated with anti-IgG-Mouse HRP at 1:5000 (81-6520, Invitrogen) for 90 minutes at RT. After two washes for 10 minutes and five washes for 5 minutes with TBST, the proteins were detected using an enhanced chemiluminescence kit for western blot (BIO-RAD) following the kit protocol. Bands were visualized using the MicroChemi 4.2 equipment (Lab Access; MicroChemi 4.2). Then, the membranes were immersed in stripping buffer (1.5% glycine, 1% SDS, 1% Tween, pH 2.2, warmed at 50 °C) for 1 hour with orbital shaking at RT. After three washes with TBST for 10 minutes each, the membranes were incubated with anti-IkB-α at 1:1000 (Sc1643, Santa Cruz), and the procedure previously described was performed. Densitometric data were obtained using the ImageJ program and expressed as phosphorylated/non-phosphorylated protein.

### 2.10 Quantitative polymerase chain reaction in skin and lymphoid tissues

The collected ear segments and MLNs were stored in DNA/RNA Shield (Zymo Research, Irvine, CA, USA) at -80 °C until use. Total RNA was extracted from the ears and MLNs using the GeneJET RNA Purification kit (Thermo Scientific, Waltham, MA, USA) following the manufacturer’s instructions and assessed for quality and concentration using a NanoDrop 2000 (Thermo Scientific). mRNA was reverse transcribed to cDNA with the Maxima First Strand cDNA Synthesis kit (Thermo Scientific). Real-time quantitative PCR (qPCR) was performed using the Maxima SYBR Green/ROX qPCR Master Mix 2× (Thermo Scientific) in a StepOne Real-Time PCR system (Thermo Scientific). The oligonucleotide sequences used are listed in **Table 1**. Each transcript level was normalized to β-actin (*Actb*) following the 2^−ΔΔCt^ method (38).

**Table 1:** Oligonucleotide sequences for this study.

Gene	Oligonucleotide	Accession number
*Tbx21* (*Tbet*)	Fw: TCCAAGTTCAACCAGCACCA Rv: ATAAGCGGTTCCCTGGCATA	NM_001107043.1
*Gata3*	Fw: AGAAGGCAGGGAGTGTGTGA Rv: TTAGCGTTCCTCCTCCAGAG	NM_133293.1
*Rorc*	Fw: GCAGCAACGGGAACAAGTAG Rv: GGGCTATACTCAAGGTGGCA	NM_001427272.2
*Foxp3*	Fw: CGGGAGAGTTTCTCAAGCAC Rv: CACAGGTGGAGCTTTTGTCA	NM_001108250.1
*Il4*	Fw: CACCTTGCTGTCACCCTGTT Rv: ACATCTCGGTGCATGGAGTC	NM_201270.1
*Il17a*	Fw: CTGCTACTGAACCTGGAGGCTA Rv: AGGGTGAAGTGGAACGGTTG	NM_001106897.1
*Il10*	Fw: TGGCTCAGCACTGCTATGTT Rv: TTGTCCAGCTGGTCCTTCTT	NM_012854.2
*Tgfb1*	Fw: GACTCTCCACCTGCAAGACCAT Rv: CGGGTGACTTCTTTGGCGTA	NM_021578.2
*Actb*	Fw: GTCGTACCACTGGCATTGTG Rv: GCTGTGGTGGTGAAGCTGTA	NM_031144.3

### 2.11 DNA extraction and qPCR for bacterial load in skin and feces

Ear samples (100–200 mg) were excised and stored in DNA/RNA Shield (Zymo Research) at -80 °C until further use. Before DNA extraction, the samples were homogenized in PBS using a basic Ultra-Turrax Homogenizer system (Ika, Staufen, Germany). Genomic DNA was obtained using the Quick-DNA Fungal/Bacterial Miniprep kit (Zymo Research) following the manufacturer’s instructions and verified for quality and concentration using a NanoDrop 2000 (Thermo Scientific). Quantitative real-time PCR was carried out with the Maxima SYBR Green/ROX qPCR Master Mix 2× (Thermo Scientific) in a StepOne Real-Time PCR system (Thermo Scientific) using the oligonucleotides listed in **Table 2**. Absolute quantification was established with a standard curve of 10-fold serial dilutions of the plasmids pTZ57R-FemA-SE and pTZ57R-FemA-SA with the *femA* cloned amplicon to *Staphylococcus epidermidis* and *S. aureus*, respectively, as previously described by (40).

**Table 2:** Oligonucleotides sequences for evaluation of skin and fecal bacteria.

Microorganisms	Oligonucleotide	References
*S. epidermidis femA*	Fw: CAACTCGATGCAAATCAGCAA Rv: GAACCGCATAGCTCCCTGC	(39, 40)
*S. aureus femA*	Fw: TGCCTTTACAGATAGCATGCCA Rv: AGTAAGTAAGCAAGCTGCAATGACC	(39, 40)
Firmicutes	Fw: GGAGYATGTGGTTTAATTCGAAGCA Rv: AGCTGACGACAACCATGCAC	(41)
Bacteroidetes	Fw: GGARCATGTGGTTTAATTCGATGAT Rv: AGCTGACGACAACCATGCAG	(41)
Proteobacteria	Fw: CATGACGTTACCCGCAGAAGAAG Rv: CTCTACGAGACTCAAGCTTGC	(42)
Actinobacteria	Fw: GADACYGCCGGGGTYAACT Rv: TCWGCGATTACTAGCGAC	(43)
*Lactobacillus*	Fw: GCAGCAGTAGGGAATCTTCCA Rv: GCATTYCACCGCTACACATG	(40)
*Bifidobacterium*	Fw: GATTCTGGCTCAGGATGAACGC Rv: CTGATAGGACGCGACCCCAT	(40)

For the evaluation of bacterial phyla and genera, fecal samples were collected, and DNA was extracted using the E.Z.N.A. Stool DNA kit (Omega Bio-Tek, Norcross, GA, USA), following the manufacturer’s protocol. DNA quality and quantity were assessed using the NanoDrop 2000 (Thermo Scientific). The samples were then analyzed for Firmicutes, Bacteroidetes, Proteobacteria, Actinobacteria, *Lactobacillus*, and *Bifidobacterium* by amplifying the *16S* rRNA gene with specific oligonucleotides (**Table 2**). qPCR was performed using the Maxima SYBR Green/ROX qPCR Master Mix 2× (Thermo Scientific) in a StepOne Real-Time PCR system (Thermo Scientific). For absolute quantification, amplicons for each phylum were cloned into the InsTAclone PCR Cloning kit (Thermo Scientific) in *Escherichia coli* DH5α to generate the plasmids pTZ57R-Firmicutes, pTZ57R-Bacteroidetes, pTZ57R-Proteobacteria, and pTZ57R-Actinobacteria; those for the genera *Lactobacillus* and *Bifidobacterium* were cloned using the pGEM T-Easy Vector System (Promega, Madison, WI, USA) as previously described (44). Plasmid isolation was performed using the PureYield Plasmid Miniprep System (Promega). Serial 10-fold dilutions were used as templates to establish a standard curve for the *16S* rRNA gene of each phylum and genus.

### 2.12 Quantification of SCFAs in cecal content

The SCFA concentration was determined as previously described (40, 45). Briefly, 100 mg of cecal contents were homogenized by vortexing in 1 mL of deionized acidified water, and centrifuged at 16,000 xg for 10 minutes at 4 °C. Supernatants were collected and filtered through a 0.22 µm filter, and then samples were measured in a gas chromatograph 6850 Network GC System (Agilent Technologies, Santa Clara, CA, USA) with an HP-5MS column coupled to a mass spectrometer 5975C VL MSD with a triple-axis detector (Agilent). The temperature in the oven was set at 100 to 240 °C with an increment rate of 15 °C/minute. The temperature of the injector and detector was set at 270 °C, and helium was used as the carrier gas at 1.5 mL/minute. The concentrations of acetic, propionic and butyric acids were determined using a standard curve of the WSFA-2 standard (Sigma Aldrich).

### 2.13 Chemical characterization of agave fructans

The AFs used in this study were obtained from *A. tequilana* Weber var. azul (Enature, Zapopan Jalisco) and the CI was used as a standard for the chemical analysis (Sigma, St. Louis, MO, USA). The functional groups of fructans were identified using the Fourier Transformed Infrared-Attenuated Total Reflection (FTIR-ATR) and ^13^C -Nuclear Magnetic Resonance (NMR) spectra, the DP was determined by Matrix-Assisted Laser Desorption/Ionization Time of Flight Mass Spectrometry (MALDI-TOF-MS), and the molecular weight (MW) was determined using ^1^H-NMR spectra. For FTIR-ATR analysis, a Nicolet Magna IR 550 spectrophotometer (Waltham, Massachusetts, USA) was used in attenuated total reflectance (ATR) mode at a wavelength scan from 550 to 4000 cm^−^¹. For MALDI-TOF-MS analysis, the m/z ratio was determined using an Autoflex maX device (Bruker Daltonics GmBH, Bremen, Germany) in positive mode. The spectrum was acquired within the range of 400 to 8000 m/z. The sample was dissolved in water and mixed with 2,5-dihydroxybenzoic acid (DHB) as a matrix. To determine the DP using this analysis, the general formula for fructans: DP = n-fructose + 1-glucose was considered. For proton (^1^H) and carbon (^13^C) NMR analysis, the samples were analyzed in a Bruker Avance III HD 400 MHz spectrometer (Billerica, Massachusetts, USA), at 25 °C using deuterated water (D_2_O) and deuterated dimethyl sulfoxide-d6 (DMSO-d_6_) as solvents, respectively. To define the MW of fructans using the ^1^H-NMR spectrum, the anomeric proton of α-glucose in the chain was identified at 5.44 ppm, and its integral was fixed at a value of 1. The rest of the signals attributed to the glucose and fructose units were present between a chemical shift range of 4.40 and 3.30 ppm. A single integral was performed on this region of the spectrum and correlated with the anomeric proton of α-glucose, resulting in the number of protons in the rest of the chain. This integral from 4.40 to 3.30 ppm was defined as X. With this data, the MW was calculated using the following formula: MW = (DP-1) x 162.145 + 180.16, considering DP = ((X − 6)/7) + 1, according to reference (46).

### 2.14 Statistical analysis

Results are presented as mean ± standard error, and statistical comparisons of different groups were performed using one-way or two-way ANOVA followed by Dunnett’s *post hoc* test for comparison of all groups against the AD group. Data analysis was performed using GraphPad Prism 7.0 software (GraphPad Software, Inc.). Statistical significance was set at P < 0.05.

## 3 Results

### 3.1 Agave fructans administration reduces the acute inflammatory reaction in AD-skin lesions

In experimental AD, the ear thickness is considered an indicator of skin inflammation in lesional areas (47). Thus, to assess the inflammatory response after allergen challenge, we analyzed the change in the right ear thickness compared to that of the left ear in each animal at 1, 6 and 24 hours after applying the DNCB on day 36. As shown in **Figure 1B**, all animals that were repeatedly challenged with DNCB presented ear inflammation before DNCB application on day 36 (0 hours), which was the result of a sustained cutaneous inflammatory response to the allergen. After DNCB application, a significant increase in ear thickness was shown in AD animals as compared to the control group (P < 0.0001). Ear swelling in AD animals peaked at 1 hour, slightly decreased at 6 hours and it was sustained until 24 hours. However, animals AFs administrated at dose of 0.1 g/kg reduced in 34% the acute inflammatory reaction (1 hour) induced by DNCB in comparation to AD group (P < 0.0001). A significant reduction (47%) in ear swelling was also observed in rats treated with cetirizine 1 hour after DNCB application. No significant changes were observed in animals administered with higher doses of AFs at 1-, 6- or 24-hours post-challenge.

Ear weight was assessed as an additional measure of tissue edema and inflammation. One hour after the DNCB challenge, ear edema in the AD group increased 3.5-fold compared with controls (**Figure 1C**). Strikingly, animals administered with AFs at 0.1 g/kg presented a 43% reduction in tissue edema (P < 0.05), which is in accordance with the anti-inflammatory effect reported by ear thickness. Treatment with cetirizine or higher doses of AFs did not significantly change ear edema induced by DNCB application, although a slight reduction was observed in the CET group.

To investigate the underlying mechanisms, NF-κB signaling was evaluated by measuring IκBα phosphorylation in ear tissue (**Figure 1D**). AD animals showed a 2.4-fold increase in the phosphorylation of IκBα compared with controls. In animals treated with AFs at dose of 0.1 and 1 g/kg, IκBα phosphorylation decreased by 54% and 63%, which reached an inhibitory effect similar to the antihistamine cetirizine group (63% of reduction vs. AD, P < 0.01) and similar values than those of the control group. No-significant changes were observed in animals treated with the highest dose of AFs (AF5 group). Altogether, these results suggest that the anti-inflammatory effect on AD lesions induced by the oral administration of low doses of AFs is mediated by the inhibition of the NF-κB inflammatory pathway.

### 3.2 Agave fructans administration does not modify pruritus

An important feature in AD is the intense cutaneous itch, which induces scratching of the affected area and exacerbation of skin lesions. Activated keratinocytes and immune cells release diverse cytokines and growth factors that stimulate sensory neurons to transmit the sensations of itching (48). In our experimental model, animals with AD exhibited an average of 69 and 53 scratching events, 10 min after DNCB application on days 22 and 36, respectively; compared with 8 and 2 events in control animals (**Figure 1E**). This entails a 7-fold and 25-fold increase in scratching frequency in animals induced by repeated DNCB application. No significant changes were observed in scratching frequency of the control and AD groups between days 22 and 36, as the rats of both groups were administered with water. Animals treated with cetirizine decreased scratching events by 43% on day 26 compared to day 22 (P < 0.01), indicating the anti-pruritic effect of the antihistamine treatment. In contrast, oral AFs administration did not modify scratching frequency after 13 days of treatment at any tested dose.

### 3.3 Agave fructans administration reduces serum total IgE levels

Serum IgE levels were measured in samples obtained 24 hours post-DNCB challenge on day 36. As shown in **Figure 1F**, animals in the AD group tripled the level of total IgE in serum as compared to the level in control animals (1577 ng/mL vs. 553 ng/mL, P < 0.05). Total IgE level was significantly reduced in AD-induced rats that were administered with AFs at 0.1 g/Kg (731 ng/mL, P < 0.05), showing similar values to those of the control group (P = 0.052). As reported to inflammation, higher doses of AFs did not have a significant effect on IgE production.

### 3.4 Agave fructans administration down-regulates epidermal thickening and mast cell and eosinophil infiltration

**Figure 2:**
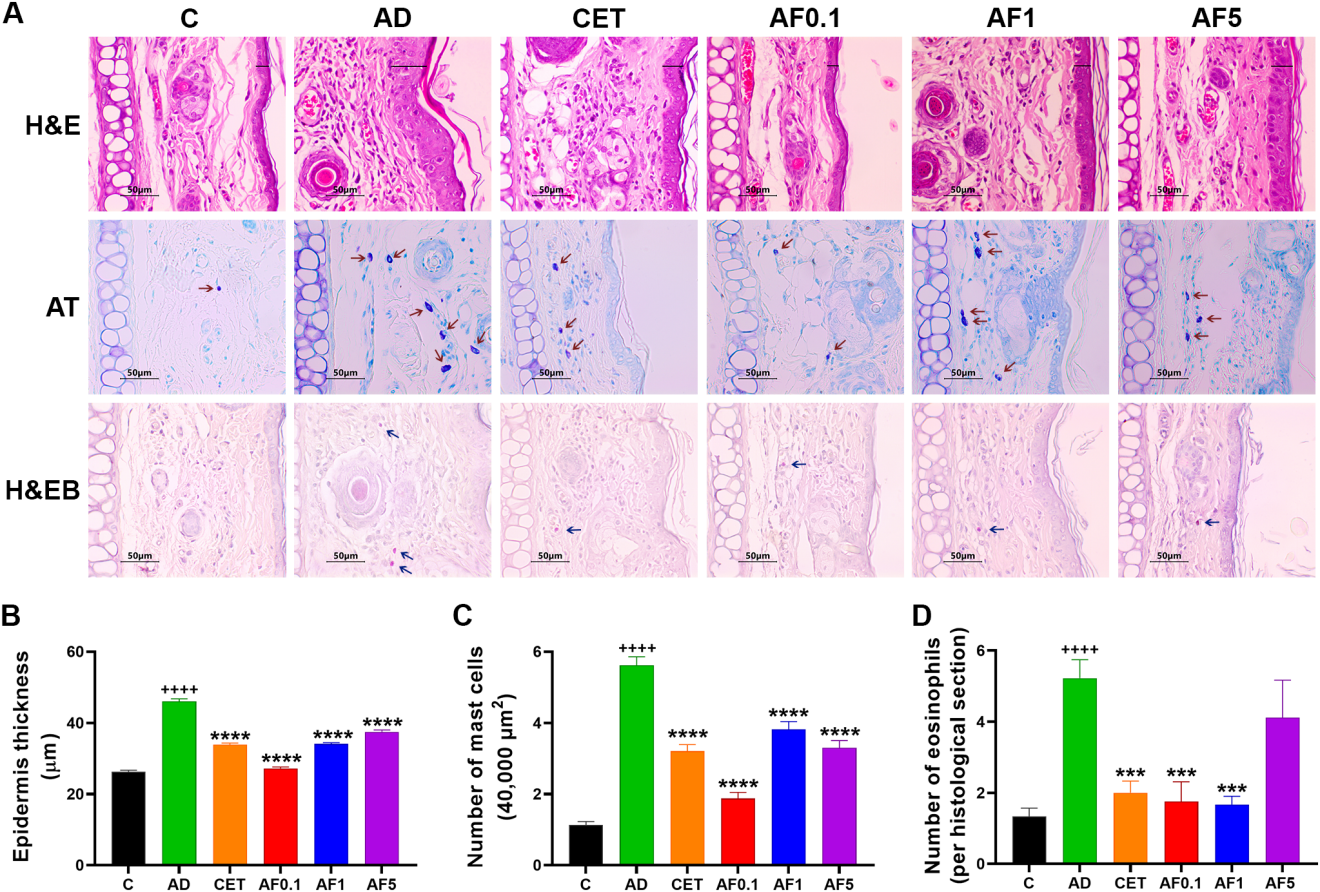
Agave fructans effect on inflammatory cell recruitment and epidermal thickness in ear tissue. **(A)** Representative images of stained ear sections from experimental groups to illustrate changes in dermal inflammatory cells density and epidermal thickness. **(B)** Epidermis thickness in ear lesional tissue; n=3 rats/group, 3 slices/rat, 6 micrographs/slice and 3 measurements/micrograph. **(C)** Number of dermal mast cells; n=3 rats/group, 3 slices/rat, 3 areas/slice. **(D)** Eosinophil infiltration in the dermis of lesional skin; n=3 rats/group, 3 slices/rat. All morphometric assessments were performed in a blinded manner by two independent researchers. ++++ P < 0.0001 vs. C; ***P < 0.001; ****P < 0.0001 vs. AD.

To determine the effect of AFs treatment on tissue damage, epidermal thickness and the number of dermal mast cells and eosinophils in lesional skin were determined by hematoxylin/eosin, toluidine blue and hematoxylin/erythrosin B staining on histological sections of the ear obtained 24 hours after the DNCB challenge. **Figure 2A** shows representative micrographs of stained ear sections from control and AD animals, with or without treatment. Morphometric analysis revealed that the thickness of epidermis in AD animals increased 1.7-fold (P < 0.0001 vs. the control group), and this increment was significantly reduced with all treatments (**Figure 2B**). Epidermal thickness was 28%, 41%, 26% and 19% lower in animals that received cetirizine or increasing doses of AFs than in animals with AD and without treatments. The effect of AFs administration was not dose-dependent, as only the rats of AF0.1 group presented epidermal thickness in lesional areas (27 µm) nearly identical to that of control animals (26 µm).

Concerning inflammatory cells, the number of mast cells present in rat dermis was prominently higher than that of eosinophils (**Figure 2A**), so mast cells were counted in a defined dermal area and eosinophils in the dermis of the entire histological section (**Figures 2C, D**). Animals with AD significantly increased the number of both inflammatory cells in the dermis compared to control animals (P < 0.0001). The increased amounts of dermal mast cells and eosinophils caused by repeated DNCB application were significantly reduced by the oral administration of cetirizine (42% for mast cells and 61% for eosinophils) and AFs at 0.1g/kg (67% for both types of cells) and 1g/kg (32% for mast cells and 69% for eosinophils). The dose of 5 g/kg of AFs only significantly reduced the mast cell hyperplasia (42%, P < 0.0001). Outstanding, the animals treated with the lowest dose of AFs showed dermal ​​inflammatory cell values closest to those of the control rats.

### 3.5 Agave fructans administration reduces the inflammatory response type-2 and -17, and *Staphylococcus aureus* colonization in AD-like lesions

**Figure 3:**
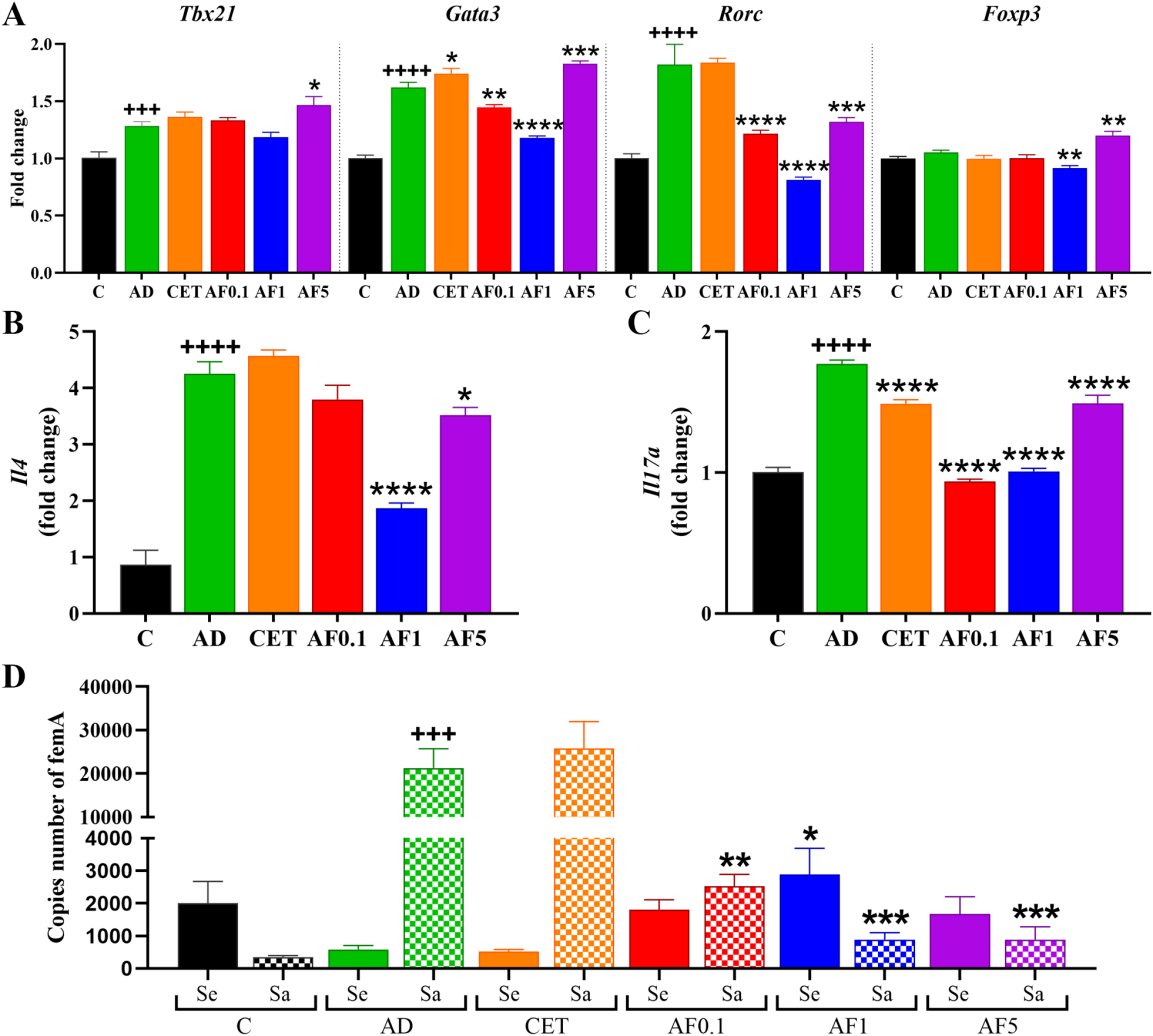
Agave fructans effect on the expression of differentiation master factor and cytokines and on *S. aureus* and *S. epidermidis* colonization in ear tissue. mRNA expression of **(A)** the transcription factors *Tbx21*, *Gata3*, *Rorc* and *Foxp3*, and the cytokines **(B)***Il4* and **(C)***Il17a*, in lesional tissue 24 hours after DNCB challenge; n=4 rats, samples analyzed in duplicate. **(D)** Amount of *S. epidermidis* and *S. aureus* in the lesional skin of animals 24 hours after DNCB application; n=3 rats, samples analyzed in duplicate. +++P < 0.001; ++++ P < 0.0001 vs. C; *P < 0.05; **P < 0.01; ***P < 0.001; ****P < 0.0001 vs. AD.

The mRNA expression levels of *Tbx21*, *Gata3*, *Rorc*, and *Foxp3* were quantified in lesional skin, as they are key factors in the differentiation of effector cells involved in type-1, -2, -17, and regulatory immune responses, which are key to the establishment of the AD model (49, 50). Expression level of *Tbx21, Gata3* and *Rorc* mRNA was significantly increased in the AD group compared with the control group, by 20%, 66% and 80% (**Figure 3A**). Regarding treatment effects, *Tbx21* expression remained unchanged in CET, AF0.1, and AF1 groups, and a significant but slight upregulation was observed in the AF5 group (P < 0.05). Oral administration of AFs at 0.1 and 1 g/kg significantly reduced *Gata3* mRNA levels with respect to the AD group. However, the treatment with cetirizine or AFs at doses of 5 g/kg did not have an ameliorative effect on the *Gata3* expression level; on the contrary, it was slightly higher than that of the AD group. In relation to *Rorc* mRNA level, AF0.1, AF1, and AF5 groups had significant down-expression as compared to those of AD animals, while cetirizine administration did not change *Rorc* mRNA expression in lesional tissue. Finally, although *Foxp3* mRNA expression was unchanged in AD, CET and AF0.1 groups, it was discreetly but significantly reduced with fructan administration at 1g/kg. Conversely, administration of AFs at 5 g/kg/day showed significantly increased levels of *Foxp3* mRNA.

As immune responses type-2 and -17 are strongly implicated in the development of AD (51) and considering that we found an increased expression of *Gata3* and *Rorc* mRNA in our AD model, we next explored two representative mRNA cytokines, *Il4* and *Il17a*, in AD lesion skin in treated groups. As shown in **Figures 3B, C**, *Il4* and *Il17a* mRNA were significantly elevated 5.2- and 1.7-fold in the AD group when compared with the levels in the control group. Cetirizine had a significant reduction of 17% just in the expression de *Il17a* mRNA. Furthermore, treatment with AFs presented a modulatory effect on the expression of these cytokines since AF0.1 shown a tendency to reduce *Il4* in 11% and significantly *Il17a* in 47%; and, although it was not in a doses-dependent manner, treatment with AFs at 1 and 5 g/kg/day reduced significantly the mRNA expression in 57% and 16% for *Il4* and 41% and 17% for *Il17a*, respectively. These results suggest that AF administration has a positive effect on mitigating the allergic inflammatory process by regulating the expression of key inflammatory genes.

AD is also characterized by reduced microbial skin diversity (*i.e.*, dysbiosis), which allows the colonization and proliferation of pathogens such as *S. aureus* (52). Skin from control animals presented 5.7-fold more abundance of *S. epidermidis* than *S. aureus*, while the relation was inverted in AD animals, with 36.4-fold more *S. aureus* than *S. epidermidis* (**Figure 3D**). Then, to determine the effect of AFs treatment on staphylococci colonization, we evaluated the bacterial load of *S. epidermidis* and *S. aureus* in the lesional skin of experimental groups. Although the copy number of the *femA* gene of *S. epidermidis* remained unchanged among the CET, AF0.1 and AF5 groups, the AF1 group had a significant increase relative to the control group (P < 0.05). The bacterial load of *S. aureus* remained unchanged with cetirizine treatment. Remarkably, AFs treatment significantly reduced the colonization of *S. aureus* in 88%, 95% and 95% in groups AF0.1, AF1 and AF5, respectively. Then, the alleviating effects of AFs intake in AD appear to be closely associated with the diminution of *S. aureus* colonization, a key driver of the AD exacerbation (11).

### 3.6 Agave fructans administration induces a regulatory immune profile on MLN and modifies the abundance of some commensal bacterial fila and genera

**Figure 4:**
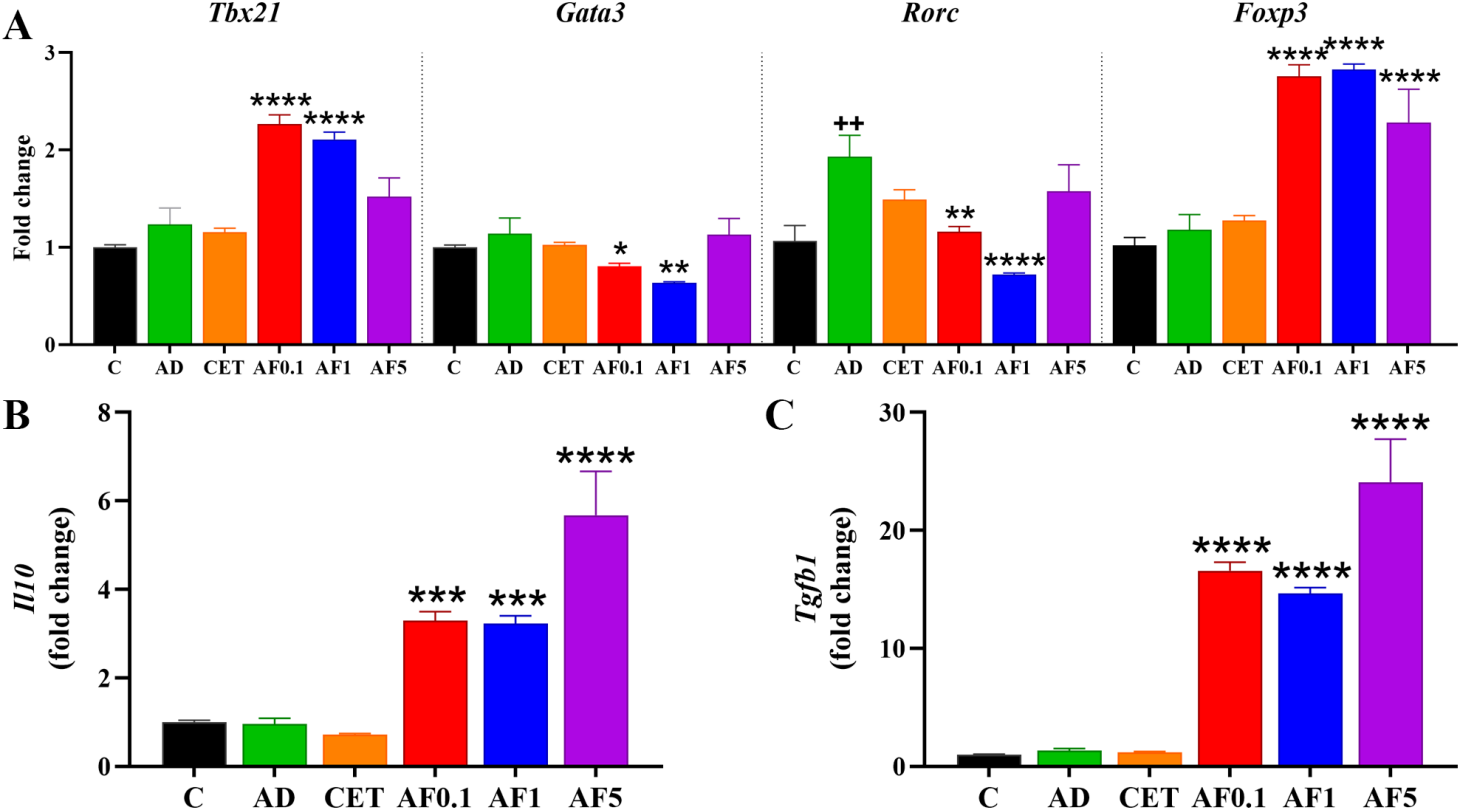
Agave fructans effect on the expression of differentiation master factor and cytokines in lymph mesenteric nodes. mRNA expression of **(A)** the transcription factors *Tbx21*, *Gata3*, *Rorc* and *Foxp3*, and the cytokines **(B)***Il10* and **(C)***Tgfb1*, in lymph mesenteric nodes collected 24 hours after allergen exposure; n=4 rats, samples analyzed in duplicate. ++P < 0.01; vs. C; *P < 0.05; **P < 0.01; ***P < 0.001; ****P < 0.0001 vs. AD.

As with other non-digestible fibers, fructans can be fermented by commensal bacteria to generate metabolites with immunomodulatory effects on mucosal immunity (53). It is important to highlight that oral administration of AFs over a 13-day period (days 24 to 36) did not alter body weight in rats, regardless of the dose (**Supplementary Figure S1B**). To determine whether AFs administration modifies the intestinal immune environment, we determined transcriptional factors in MLN from experimental groups (**Figure 4A**). There were no differences in the expression of *Tbx21*, *Gata3* and *Foxp3* mRNA in the group AD; however, the level of *Rorc* mRNA was significantly higher in the AD group than that of the control by 79%. Besides, AFs at doses of 0.1 and 1 g/kg/day induced significant increments in levels of *Tbx21* and *Foxp3* mRNA of 1.8-, 1.7-, 2.4- and 2.5-fold, respectively. Moreover, doses of 5 g/kg/day maintained significantly upregulated *Foxp3* mRNA expression. Interestingly, *Gata3* and *Rorc* were significantly downregulated when animals received 0.1 and 1 g/kg/day of AFs as compared to the AD group. Conversely, the AF5 group did not show differences when compared to the AD group. As prominent expression of *Foxp3* transcription factor in T regulatory (Treg) profile was observed, next we measured the expression levels of *Il10* and *Tgfb1* mRNA (**Figures 4B, C**). There were 3.5, 3.5 and 6.2-fold higher transcriptional levels of *Il10* mRNA in animals that received 0.1, 1 and 5 g/kg/day of AFs. Similarly, the expression of *Tgfb1* mRNA was 12.6-, 11.2- and 18.5-fold upregulated after treatments with the respective doses of AFs. Taken together, these findings suggest that promoting intestinal homeostasis and an immunoregulatory environment with AFs may lead to favorable distant effects on skin inflammation.

**Figure 5:**
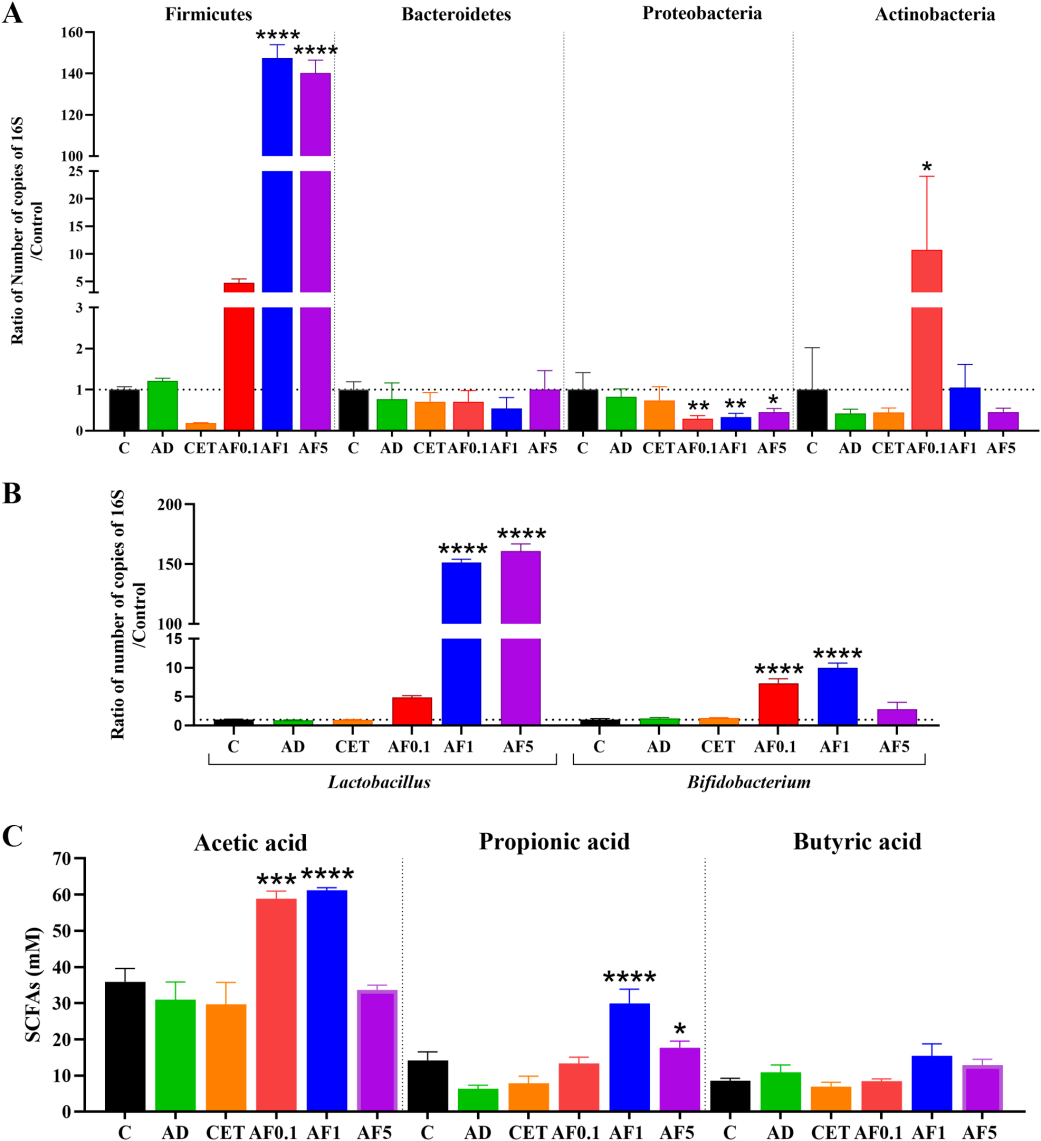
Agave fructans effect on commensal bacterial abundance and cecal SCFAs levels. Relative abundance of **(A)** bacterial phyla Firmicutes, Bacteroidetes, Proteobacteria, Actinobacteria, and **(B)** genera *Lactobacillus* and *Bifidobacterium* in fecal sample collected from animals following 13 days of oral agave fructans administration; n=3 rats, samples analyzed in duplicate. **(C)** Concentrations of acetate, propionate and butyrate in cecal contents obtained from the same animals after the 13-day treatment period; n=4 rats. *P < 0.05; **P < 0.01; ***P < 0.001; ****P < 0.0001 vs. AD.

Pathogenesis and severity of AD are frequently associated with genetic and environmental factors. In recent years, the impact of gut microbiota on a crosstalk between intestinal and skin homeostasis has gained clinical importance. Thus, the therapeutic approaches also involve probiotics and prebiotics administered orally (54, 55). Previous studies have demonstrated that inulin-type fructans exert prebiotic properties, since when orally administered they favor the abundance of Firmicutes and Actinobacteria (56, 57). To evaluate possible changes induced by AFs intake in the main phyla in gut microbiota, we performed absolute qPCR to determine the copy number of the *16S* rRNA gene in fecal DNA (**Figure 5A**). The AD group had similar bacterial abundance of the four analyzed phyla than that of control group. Notably, in groups that received AFs, abundance of Firmicutes was 3.9-, 122.9- and 116.8-fold higher in the AF0.1, AF1 and AF5 groups, respectively, in comparison to that in the AD group (P < 0.0001 AF1 and AF5 vs. AD). Besides, Proteobacteria showed a significant decrease of 75, 62 and 50% respectively, in animals treated with AFs at 0.1, 1 and 5 g/kg/day. Finally, in the animals that received 0.1 g/kg/day of AFs the amount of Actinobacteria rose 26.5-fold compared to AD group. The abundance of *Bacteroidetes* did not significantly differ among all the groups. Then, we evaluated the abundance of the two main genera of lactic acid bacteria. As shown in **Figure 5B**, there was a similar abundance of *Lactobacillus* and *Bifidobacterium* in feces from control, AD and CET animals. Nevertheless, lactobacilli increased 6-, 189.3- and 201-fold in rats administered with 0.1, 1 and 5 g/kg/day of AFs (P < 0.0001 AF1 and AF5 vs. AD), while the abundance of bifidobacteria was only significantly 6.1- and 8.2-fold up-regulated with the oral intake of 0.1 and 1 g/kg/day of AFs. These results strongly provide evidence that AFs have positive effects on AD via changes in the intestinal bacterial community.

The anti-inflammatory effect of non-digestible fibers is partly mediated by SCFAs produced during gut microbial fermentation (55). Thus, we analyzed the SCFAs in cecal content from our experimental animals. As shown in **Figure 5C**, there is no change in the amount of acetic, propionic and butyric acids in cecal samples from control, AD or CET groups. Importantly, levels of acetic acid were duplicated in the cecum of animals treated with AFs at 0.1 and 1 g/kg/day. Besides, propionic acid was 4.7-fold and 2.8-fold increased when rats were orally supplemented with 1 and 5 g/kg/day of AFs. None of the used AF doses modified the cecal level of butyric acid. Then, it is feasible that production of SCFAs may prompt the increment of *Il10* and *Tgfb* mRNA expression to dampen inflammation of the skin and favor the alleviation of AD symptoms.

### 3.7 Chemical structure of agave fructans

**Figure 6:**
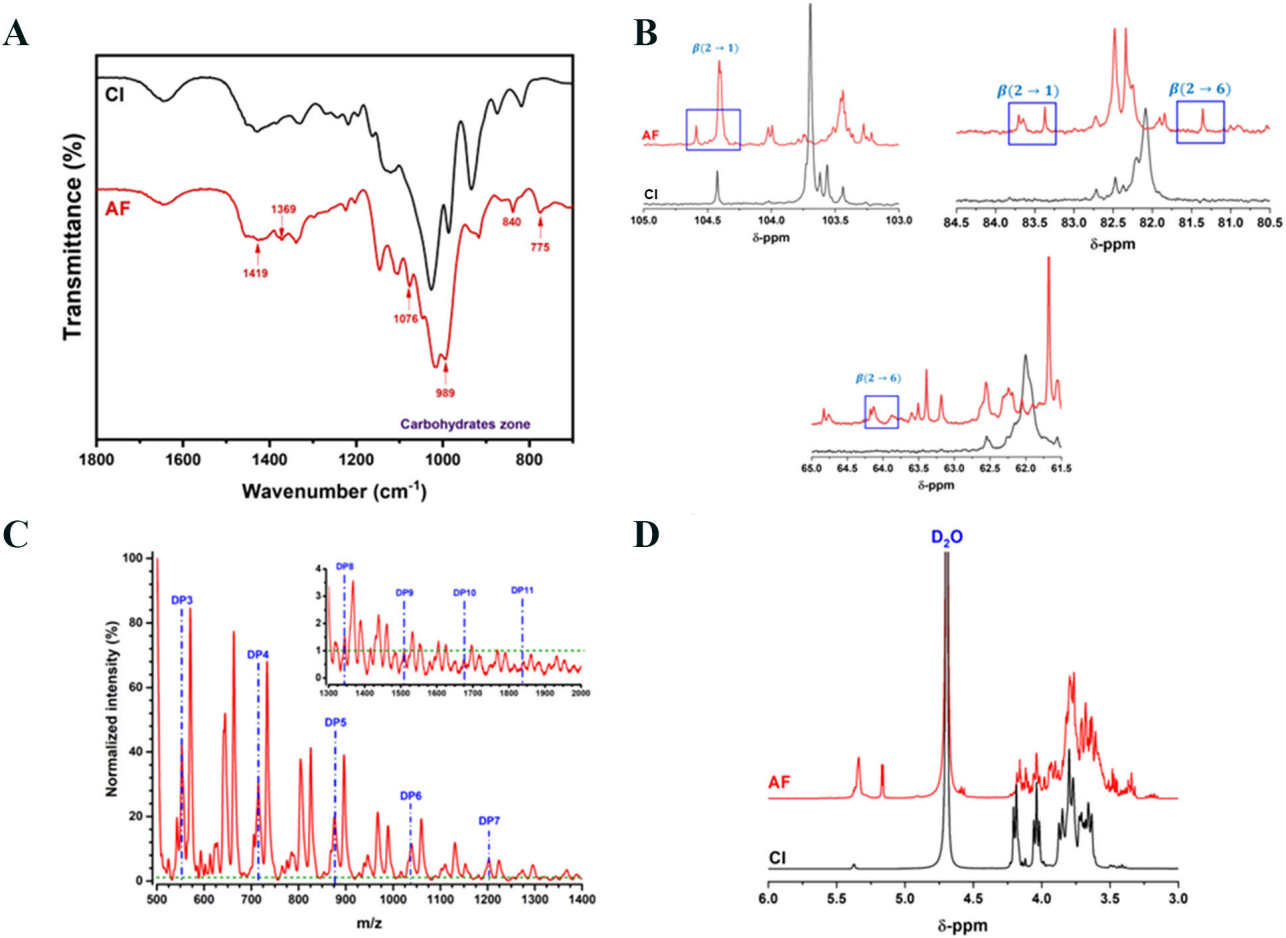
Chemical characterization of agave fructans. **(A)** Fourier Transform Infrared-Attenuated Total Reflection spectra **(B)**^13^C-Nuclear Magnetic Resonance (NMR) in DMSO-d_6_ spectra, **(C)** Matrix-Assisted Laser Desorption/Ionization Time of Flight Mass Spectrometry spectra, and **(D)**^1^H-NMR spectra, of agave fructans (AF) used in the study. Chicory inulin (CI) was used as a standard for FTIR and NMR analysis.

We sought to assess whether the chemical structure of the commercial AFs used in this study corresponded to that previously reported for agavins (**Supplementary Figure S2A**). **Figure 6A** shows the FTIR-ATR spectra of fructans, and the main peaks were found between 1800 and 700 cm^-1^, which is a characteristic of polysaccharides (27, 58–61). A small but perceptible band was observed at 1419 cm^-1^, which can be assigned to the deformation of CH_2_-OH on the fructose ring. A slight peak was observed at 1369 cm^-1^, which could be assigned to C-O-C bonds between monomers and can be confirmed with intensive peaks observed at 989 cm^-1^; these peaks are associated with glycosidic bonds present in the polysaccharide structures. Additionally, the peak at 1076 cm^-1^ could be assigned to C-O and C-C stretching vibrations of the furanose ring (58–60). A small peak was observed at 775 cm^-1^, whose small absorptions are associated with vibrations related to the configuration of the fructose molecule (major element in agavins) (60). An important point is that AFs showed the presence of different peaks associated with a high concentration of groups assigned to fructose, which suggests that the 775 cm^-1^ peak could be related with β (2→6) bonds characteristic of agavin structure. As previously reported, the area below 930 cm^-1^ (from 930 to about 700 cm^-1^) is very characteristic of vibrations originating from the anomeric region of carbohydrates or from C–H and C–C deformation. Even small changes in vibrations from this region (such as 840 cm^-1^) usually indicate strong modifications/differences in the glycosidic bonds (62–64).

To further confirm the presence of β (2→6) bonds, we performed ^13^C-NMR analysis (65). The AFs spectrum revealed an overlapping of signals, which could be attributed to the presence of high amounts of both types of links and a branched structure (**Supplementary Figure S2B**). However, it was possible to see important signals in a specific range (**Figure 6B**). Thus, signals were observed at δ104.25 ppm and δ104.31 ppm, which could indicate the presence of β-D-Fruf residues, and the signal at δ104.52 ppm was related to internal β (2→1) moieties (66). The signal at δ81.35 ppm was attributed to the C-5 in the furanosyl unit substituted at O-6 in the β (2→6) bond (59, 60, 66, 67). Also, signals observed at δ83.37 and δ83.7 ppm were assigned to β (2→1) bonds in the main structure of AFs. Additionally, signals at δ64.09 ppm and δ63.98 ppm provided strong evidence of the presence of the β (2→6) bond link of D-Fru*f*- moieties (19, 66). The characteristic signals of the functional groups present in a linear chain of fructosyl-fructose with β (2→1) bond were observed at δ103.26 ppm in the CI spectrum (59, 60, 67).

The DP in fructans is directly related to their biological activity (27). **Figure 6C** shows the corresponding DP profile obtained from the AFs sample used in this study by MALDI-TOF, which revealed a DP between 3 and 11, with a decreasing trend in the amount of each fructan as DP increases, as previously described for fructans obtained from agave plants (19). This result indicates that AFs are short polysaccharides with predominant oligosaccharide content. Using ^1^H-NMR analysis (**Figure 6D**), AFs sample showed a MW average of 2800 Da. Comparing the region between 4.40 and 3.30 ppm in the ^1^H-NMR spectra of AFs and CI, a more complex set of signals was clearly visible for the former, indicative of the high branching in the chains.

## 4 Discussion

AD ranks first among skin diseases in disability-adjusted life-years, exhibiting the highest prevalence in early childhood, followed by a secondary peak in middle age (68). Recent research suggests a progressive manifestation of atopic conditions, termed the “atopic march,” characterized by the sequential development of asthma, allergic rhinitis, and food allergies in later childhood, closely linked to early-onset AD (69). These characteristics of AD, along with its progression to chronicity and the association between atopic disease onset and intestinal dysbiosis, facilitate and support the development of non-medical or non-pharmacological interventions, including lifestyle modification strategies. The intake of fermentable fibers is a dietary strategy with promising immunomodulatory effects through addressing gut dysbiosis. We demonstrated that the consumption of AFs from *A. tequilana* Weber var. azul reduces acute inflammation and edema in rat AD-like lesions induced by allergen exposure. The anti-inflammatory effect is related to the inhibition of the NF-κB inflammatory pathway in lesional tissue. Oral ingestion of AFs also decreases mast cell hyperplasia, eosinophil abundance, and epidermis thickness in ear lesions, along with lower serum total IgE levels. The predominant type-2 and -17 immune responses, as well as excessive *S. aureus* colonization in lesional tissue, are downregulated. In the intestinal microenvironment, AFs promote a regulatory prolife at MLN, increase the abundance of Firmicutes, *Lactobacillus* and *Bifidobacterium* and reduce Proteobacteria in feces, alongside higher cecal levels of acetic and propionic acids.

The chemical analysis of fructans from *A. tequilana* Weber var. azul used in this study validated the characteristic structure of agavins, with linear and branched chains of fructose connected by β (2→1) and β (2→6) fructosyl-fructose linkages (19). To assay the effect of AFs on cutaneous lesions, we chose the previously reported Wistar rat model of systemic sensitization using Th2 adjuvants followed by repeated topical application of DNCB (33, 36). In this model, AD animals presented acute and late-phase inflammatory reactions, high serum levels of total IgE, intense skin pruritus and epidermal thickness. Lesional tissue showed high expression of transcription factors and cytokines related to type-2, type-1 and type-17 reactions, and an extensive colonization by *S. aureus*. Thus, the model allows us to evaluate key clinical symptoms and immunological mechanisms involved in human AD (70).

In recent years, there has been a growing interest in the study and application of functional foods. Diverse plant-derived polysaccharides have been reported to exert anti-inflammatory activity in skin when orally administered (71, 72). In relation to fructans, most studies focus on CI, with no work on AFs, particularly those obtained from *A. tequilana* Weber var. azul. Oral administration of CI in a murine model of oxazolone-induced AD produces a decrease in the expression level of pro-inflammatory markers in skin, such as calprotectin (S100A8 and S100A9) and IL-1β (30). We observed that oral administration of AFs decreases cutaneous acute inflammation and edema induced by allergen exposure to rats, which was related to a reduction in IkB-α phosphorylation. Phosphorylation of IKK proteins is a key factor for the translocation of NF-κB to the nucleus and the expression of inflammatory cytokines (73). Previously, it was reported that AFs, alone or combined with an organic extract from *A. tequilana*, cause a decrease in joint inflammation and a reduction in the expression of some pro-inflammatory cytokines, such as IL-1β, IL-6, TNF-α and IFN-γ in a murine systemic autoimmunity type-SLE model (74). In the present study, AFs treated AD-animals also reduced total IgE levels in serum, as previously reported with other plant-derived polysaccharides (75). IgE could induce mast cell activation through IgE surface receptors and the consequent release of inflammatory cytokines and granule mediators (76). Thus, the regulatory effect of AFs in inflamed tissues might be partly mediated through decreasing NF-kB signaling and IgE production.

Epidermal thickening and dermal infiltration by type-2 inflammatory cells, including mast cells, eosinophils, ILC2s, and TH2 cells, are specific hallmarks of AD acute cutaneous lesions (77). In the present study, we found that oral administration of AFs for 13 days improves the thickness of the epidermis and decreases the number of mast cells and eosinophils in the dermis. Consistent with our results, previous studies have shown that oral CI reduces epidermis thickness, and mast cell hyperplasia in a murine model of oxazolone-induced AD, when administered for 3 weeks (30). CI treatment has been associated with an increase in the expression of galectin-9, which is related to the inhibition of mast cell degranulation, the decrease of allergic inflammatory reactions in the skin, and the suppression of TH1 and TH17 cells (29, 78). It is known that AD is characterized by a mixed TH1, TH2 and TH17 cytokine expression, which are progressively heightened from acute to chronic lesions (10). Therefore, current AD therapies are aimed at regulating the expression of the main cytokines that contribute to pathologic changes in skin structure and barrier functions as well as immune dysregulation (79–82). In this sense, our results show that the AFs anti-inflammatory effect is associated with a reduced expression of IL-4 and IL-17 in lesional tissue. *Gata3* and *Rorc*, whose expression was also diminished by AFs administration, are key transcriptional factors implicated in the differentiation of TH2 and TH17 cells and in their ability to produce signature cytokines, such as IL-4 and IL-17, respectively (83, 84). Besides, GATA-3 and RORγt are central mediators in type-2 and type-17 inflammation (85, 86). Eosinophils are also a source of IL-17, contributing to skin inflammation in AD (87). Thus, these results confirm the immunomodulatory properties of AFs on dysregulated immune responses underlying AD. However, no anti-pruritic effect is observed in AD animals treated with AFs, although it is known that type 2 cytokines sensitize cutaneous sensory neurons, lowering their activation thresholds and fueling pruritus (88). These findings suggest that, in our AD model, pruritus is primarily mediated by pruritogenic factors other than IL-4, namely IL-22, IL-31, IL-33, substance P (SP) or thymic stromal lymphopoietin (TSLP) (89), and partially by histamine, as evidenced in the cetirizine-treated group. The IL-31 receptor is known to be expressed on sensory neurons and keratinocytes. Upon stimulation by IL-31, it promotes leukotriene B4 (LTB4) secretion in keratinocytes, which in turn induces pruritus via activation of the LTB4 type 1 receptor on sensory nerve fibers (90). SP acts on sensory neurons and stimulates mast cells through the MrgprA1 receptor, also contributing to itch (91). TSLP, produced by epidermal keratinocytes, is considered a key factor in perpetuating the itch-scratch cycle by activating C-fibers through its heterodimeric receptor or by inducing periostin secretion from keratinocytes (92, 93). IL-33 can activate mast cells, eosinophils, ILC2s, and sensory nerve fibers (93). Thus, further studies assessing epidermal levels of SP, LTB4, TSLP, IL-31, and IL-33 may help identify the primary pruritus mediators involved in experimental AD.

Lesional skin in AD is characterized by an overgrowth of *S. aureus*, which contributes to disease pathogenesis by suppressing beneficial commensal bacteria such as *S. epidermidis*, exacerbating skin barrier dysfunction, promoting type-2 inflammation, and increasing the risk of recurrent skin infections (94). Notably, rats orally administered AFs exhibited a reduction in *S. aureus* and an increase in *S. epidermidis* colonization, an effect not observed in those treated with the antihistamine cetirizine. The type-2 inflammation, particularly IL-4, is known to play a pivotal role in the adhesion of *S. aureus* to the skin by inducing fibronectin production in fibroblasts, thereby facilitating bacterial attachment to damaged tissue (95–97). Accordingly, the observed reduction in cutaneous *S. aureus* may be associated with decreased *Il4* and *Gata3* mRNA expression in the lesional skin of treated rats. Moreover, *S. epidermidis* produces selective antimicrobial peptides targeting *S. aureus* (98); thus, restoration of commensal *S. epidermidis* levels may also contribute to the suppression of *S. aureus* colonization. The post-fermentation supernatant of FOS by *S. epidermidis* inhibits *S. aureus* biofilm formation *in vitro* (99), suggesting that fructans could serve as topical prebiotics to control *S. aureus* overgrowth. Recently, in a murine model of contact hypersensitivity, it was demonstrated that scratching induces the release of SP from cutaneous neurons, which subsequently activates mast cells and maintains inflammation, but it enhances host defense against *S. aureus* (100). Whether the observed decrease in *S. aureus* colonization in AF-treated animals is attributable to the persistent scratching behavior remains to be elucidated. Supporting this hypothesis, the cetirizine-treated animals, characterized by reduced itch sensation, did not exhibit alterations in *S. aureus* abundance or in *Il4* and *Gata3* expression.

In the present study, we evaluated the anti-allergic response of three oral doses of AFs, 0.1, 1 and 5 g/kg, corresponding to approximately 18, 180 and 900 mg per day. These doses have been previously documented as biologically active and non-toxic (101, 102). Interestingly, we do not observe a dose-response effect in the anti-inflammatory and immunomodulatory activity. The low and intermediate doses showed greater efficacy, while the high dose of AFs did not modify NF-κB activation and significantly upregulated GATA3 expression in lesional tissue. The oral administration of a high-fiber diet based on CI (26% inulin, equivalent to ~1,040 mg per day) to mice exacerbates the type-2 immune response in the lung and colon (31). Similar results were observed by Han and coworkers when administering a similar amount of inulin to mice (103). In both works, the up-regulatory TH2 activity is mediated by an increase in levels of colic acid induced by microbiota. Nevertheless, lower doses of CI, such as 1% (29), ~15–20 mg per day (30) or 55 mg per day (103) reduce TH2 immune response ameliorating allergic responses when orally administered to mice. Hence, the dose of orally administered fructans may shape the nature of immunomodulatory effects at barrier surfaces, such as mucosal tissue and skin, and should be carefully considered when extrapolating findings to clinical settings. Our results reinforce the dose-dependent nature of prebiotic immunomodulation and underscore the need to define the specific doses at which each prebiotic exerts its effects. Further research into the health effects of high-dose administration of agave fructans is warranted to identify potential adverse outcomes and to elucidate possible saturation phenomena or potential changes in the intestinal absorption of nutrients that modulate the immune response.

In the context of the gut–skin axis, assessing immune profiles in MLNs is critical for understanding the immunomodulatory effects of oral fibers. Animals of the AD group displayed a dominant type-17 response, which was downregulated upon fructan administration, with a concurrent transition toward type-1 and regulatory phenotypes, with an increased expression of *Il10* and *Tgfb1*. There is increasing evidence indicating that Foxp3+ Treg cells play a fundamental role in immune tolerance and control of TH2 responses (104). Levels of IL-10 in AD are related to the severity of the pathology, since patients with severe dermatitis have low levels of IL-10 (105, 106). In this context, IL-10 produced by Breg cells directly inhibits the activation and infiltration of eosinophils into the skin (107). Similarly, CI decreases mRNA expression of RORγt and increases Foxp3 and TGF-β expression in MLNs of AD mice (29). It is inferred that AFs may regulate allergic reactions associated with AD through their positive effect on a regulatory intestinal environment. On the other hand, it has been reported that healthy mice fed with CI show a significantly TH1-skewed immune profile characterized by increased T-bet+ T cells in MLN (108). Thus, the immune modulation of AFs might be also associated with a rebalancing of the TH1/TH2 ratio, as demonstrated by the increased expression of *Tbx21* cells.

Studies suggest that the protective or beneficial effect of oral administration of prebiotics is due to the direct impact on the intestinal proliferation of beneficial bacteria and the generation of fermentation products with immunomodulatory activity, such as SCFAs (55). In an obese mice model, AFs administration modified the relative abundance of cecal Firmicutes, Bacteroidetes and Proteobacteria phyla, restoring values to those of control animals (109). In the present study, AD induction did not significantly change the gut bacterial phyla in rats, but all AD animals administered with AFs presented a significant decrease in fecal Proteobacteria, along with an increase in Firmicutes. Only the low doses of AFs induced a significant increase in the Actinobacteria phylum. This data is in accordance with the high load of *Lactobacillus* and *Bifidobacterium* in fecal samples, and the elevated amount of acetic and propionic acids in cecal content. In contrast, immunomodulatory effects of CI on AD are associated with reduced number of Firmicutes, increased levels of Bacteroidetes and of selected *Lactobacillus* species, with no change in certain bifidobacteria (29, 30), revealing that structurally distinct fibers are differentially processed and metabolized by the gut microbial community. Several studies have demonstrated the prebiotic activity of AFs on *Bifidobacterium* and *Lactobacillus* species, highlighting the impact of the fructans DP, as those with DP ranging from 3 to 22 stimulated better bifidobacterial growth (101, 110). Various studies indicate that agavins are low molecular weight, highly branched carbohydrates, with DP ranges between 4 to 10 units or 2 to 60 units, depending on the agave source, age and the chemical environment to which the molecules were exposed (19, 20, 66, 111, 112). Accordingly, the AFs used in our study presented a branched structure and a DP from 3 to 11. Newborns fed with a formula supplemented with a probiotic plus AFs increase the abundance of *Bifidobacterium* more than those fed without the fructans (23). Besides, healthy adults who consumed AFs are mainly enriched with *Bifidobacterium* species, and present a depletion of *Desulfovibrio*, a proteobacteria that colonizes the proximal intestine (113). When healthy mice were fed with a diet supplemented with AFs a significant increment in *Bifidobacterium* and *Lactobacillus* was reported in a time-dependent way, as the increase of *Bifidobacterium* was faster (24h) than that of *Lactobacillus* (42 days) (22). The animals also exhibited elevated levels of fecal acetate and propionate, but not of butyrate, which agrees with our results and those obtained when AFs are fermented using a pH-controlled anaerobic batch culture inoculated with human fecal slurries (114). It is known that butyrate is the preferred fuel source for colonocytes, propionate is mainly metabolized by the liver, while acetate is the main SCFA to enter the circulation where it can act on immune cells and peripheral tissues and potentially elicit anti-inflammatory effects (115, 116). The immunomodulatory activity of butyrate is primarily exerted in the gut by increasing colonic Treg cell levels (117). Both butyrate and propionate enhance the capacity of dendritic cells to promote extrathymic peripheral polarization toward Treg cells through histone deacetylase (HDAC) inhibition (118). Acetate influences Treg development mainly via the GPR43 receptor or HDAC inhibition, and these mechanisms are associated with significant suppression of experimental allergic airways disease and protection against food allergies (119–121). Additionally, acetate directly promotes the differentiation of B10 cells, which in turn induce the conversion of native T cells into Treg cells (122). Collectively, SCFAs shift the immune balance toward an anti-inflammatory milieu. Previous studies have demonstrated a non-linear correlation between CI intake and cecal SCFA concentrations, with levels decreasing when the inulin percentage is doubled (20% vs. 10%) (123). This trend is consistent with our observed reductions in acetate and propionate concentrations, as well as in the abundance of *Bifidobacterium* spp., an acetogenic genus (124), in the AF5 group compared to AF1. Taken together, these findings suggest that changes in intestinal microbial communities, together with the production of the immunomodulatory SCFAs acetate and propionate, may contribute to the attenuation of inflammation on lesional AD induced by AFs intake, exhibiting a non-lineal dose-response relationship.

In conclusion, this study demonstrates that agave-derived fructans exhibit anti-inflammatory, immunoregulatory, and gut microbiota-modulatory properties that may contribute to the therapeutic management of AD. Besides, this novel therapy reduces the abundance of *S. aureus*, a key driver of skin microbial dysbiosis in AD, while fostering the growth of beneficial staphylococci, such as *S. epidermidis*. Our findings provide robust evidence supporting the consideration of AFs consumption, a soluble dietary fiber derived from *A. tequilana* Weber var. azul, as a scientifically grounded strategy within the context of AD therapy. Nonetheless, the present study has certain limitations that warrant further investigation to fully elucidate the mechanisms involved and validate these findings in broader pre-clinical contexts. One of the limitations of the study is the exclusive use of male rats in the experimental design, which limits the extrapolation of findings due to known sex-based differences in immune responses; thus, future studies should address the inclusion of both male and female experimental groups with AD. Another limitation of our study is that the long-term efficacy of AFs administration in preventing new flares and relapsing lesions was not assessed, especially in relation to standard pharmacological therapies. Future research in this direction is warranted to enhance the translational relevance of the findings. Finally, quantifying fermentation-derived metabolites of AFs in the bloodstream, along with a broader analysis of the intestinal microbiota, may offer valuable insights into the underlying mechanisms. Further *in vivo* functional assays involving SCFA supplementation in AD animals might help clarify the causal relationship.

## Data Availability

The raw data supporting the conclusions of this article will be made available by the authors, without undue reservation.
