# Supplementary figures and images for: Immunomodulatory and anti-inflammatory effects of agave fructans in atopic dermatitis: gut microbiota and short-chain fatty acid implication
Source: Front Immunol. 2025 Dec 2;16:1700023. doi: 10.3389/fimmu.2025.1700023 (PMC12705406; doi:10.3389/fimmu.2025.1700023)

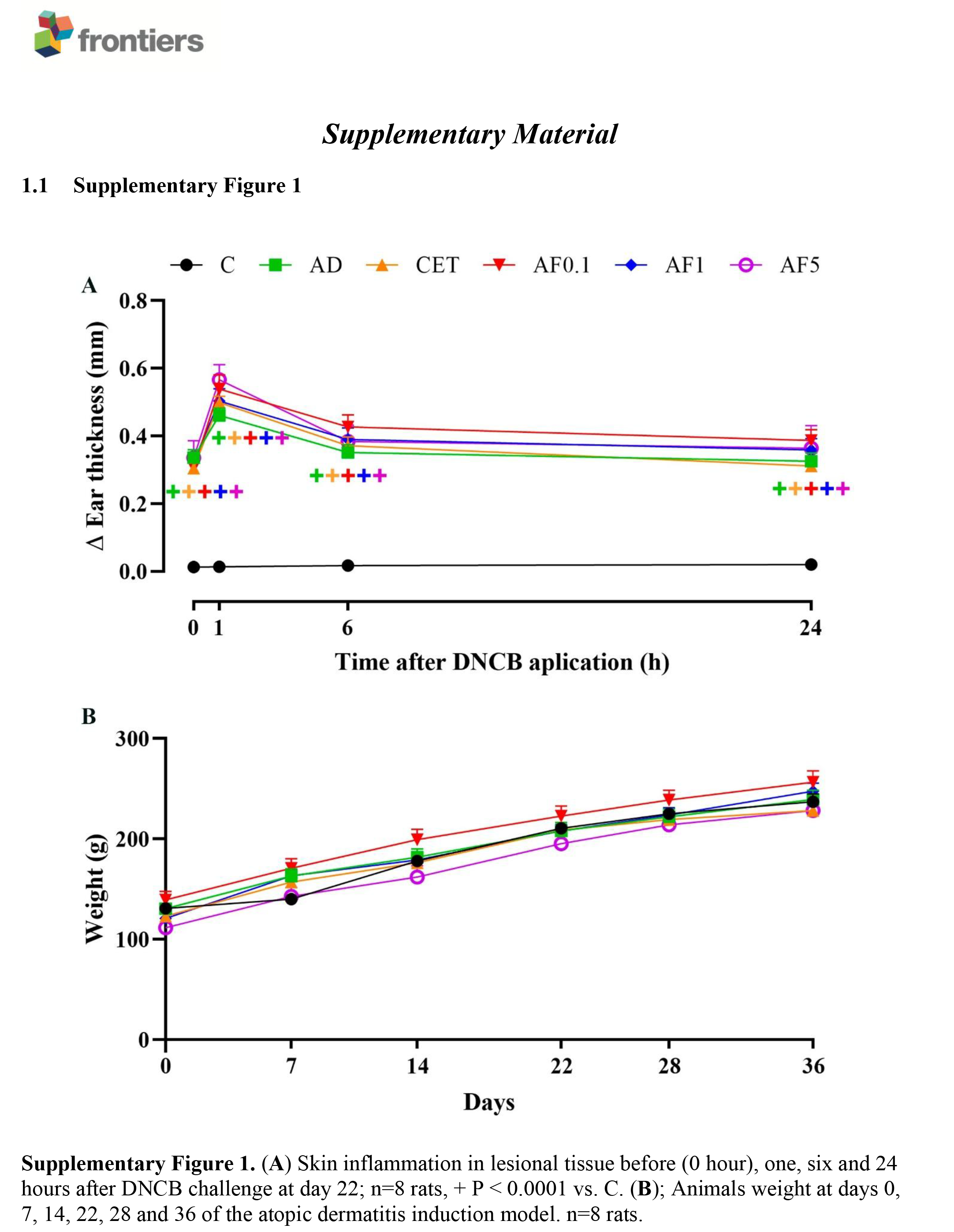

Supplement: Supplementary file 1 [file Image1.tif]

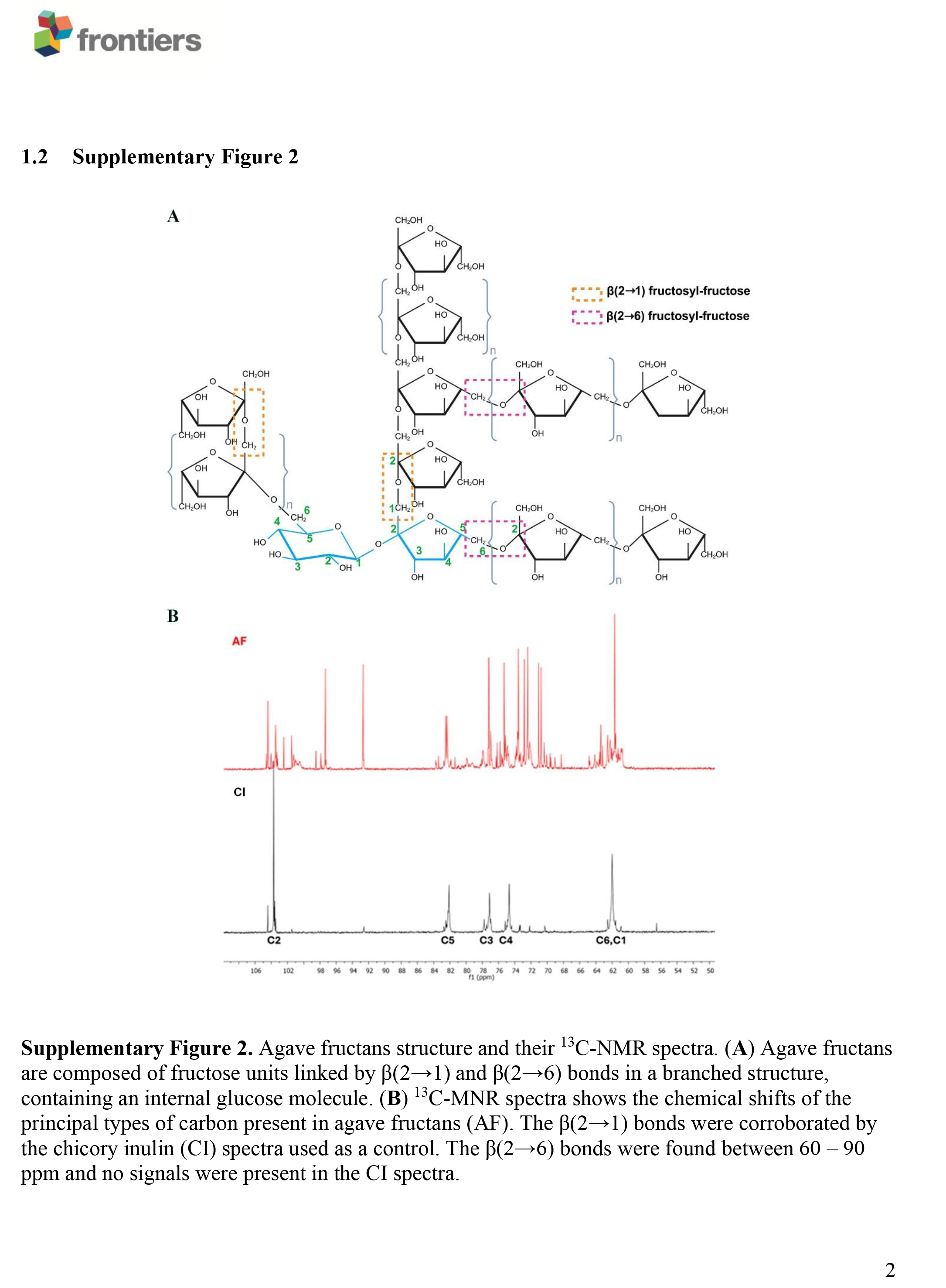

Supplement: Supplementary file 2 [file Image2.tif]
